# Wnt Ligands Differentially Regulate Toxicity and Translocation of Graphene Oxide through Different Mechanisms in *Caenorhabditis elegans*

**DOI:** 10.1038/srep39261

**Published:** 2016-12-13

**Authors:** Lingtong Zhi, Mingxia Ren, Man Qu, Hanyu Zhang, Dayong Wang

**Affiliations:** 1 Key Laboratory of Environmental Medicine Engineering in Ministry of Education, Medical School, Southeast University, Nanjing 210009, China

## Abstract

In this study, we investigated the possible involvement of Wnt signals in the control of graphene oxide (GO) toxicity using the *in vivo* assay system of *Caenorhabditis elegans*. In nematodes, the Wnt ligands, CWN-1, CWN-2, and LIN-44, were found to be involved in the control of GO toxicity. Mutation of *cwn-1* or *lin-44* gene induced a resistant property to GO toxicity and resulted in the decreased accumulation of GO in the body of nematodes, whereas mutation of *cwn-2* gene induces a susceptible property to GO toxicity and an enhanced accumulation of GO in the body of nematodes. Genetic interaction assays demonstrated that mutation of *cwn-1* or *lin-44* was able to suppress the susceptibility to GO toxicity shown in the *cwn-2* mutants. Loss-of-function mutations in all three of these Wnt ligand genes resulted in the resistance of nematodes to GO toxicity. Moreover, the Wnt ligands might differentially regulate the toxicity and translocation of GO through different mechanisms. These findings could be important in understanding the function of Wnt signals in the regulation of toxicity from environmental nanomaterials.

Graphene is a two-dimensional (2-D) carbon nanostructure. Due to the unique physical and chemical properties, the graphene family, including its derivative graphene oxide (GO), has received a lot of attention for their potential application in biosensors, bio-imaging, cancer therapy, and drug delivery^1^,^2^,^3^. Graphic nanomaterials have been predicted to overtake carbon nanotubes in commercial applications^4^. The broad spectrum of potential industrial and medical applications of graphic nanomaterials will increase the likelihood of their release into the environment. The cytoxicity of GO in inducing oxidative stress, cell division suppression, cell death, and immunotoxicity has been demonstrated by several studies^5^,^6^,^7^,^8^. Additionally, the *in vivo* studies carried out in mice or rats have suggested that the toxic effects of GO exposure at least include the induction of pulmonary toxicity and reproductive toxicity^9^,^10^,^11^,^12^. Nevertheless, the underlying molecular mechanisms for the observed GO toxicity are still largely unclear.

Up to this point, the classic animal model *Caenorhabditis elegans* has been widely used in the assessment of toxicity and toxicological studies of environmental toxicants, including the engineered nanomaterials (ENMs)^13^,^14^,^15^,^16^. *C. elegans* have the typical properties of animal models, including a short life-cycle and lifespan, transparent bodies, the ability to self-fertilize, ease of culture, and a sensitivity to environmental toxicants^13^,^17^. Using the *in vivo* assay system of *C. elegans*, it has been shown that GO exposure could result in adverse effects on the functions of both primary (such as the intestine) and secondary (such as neurons and reproductive organs) targeted organs^18^,^19^,^20^,^21^. In nematodes, the activation of oxidative stress, bioavailability, intestinal permeability, and defecation behavior may all greatly contribute to the induction of GO toxicity^19^,^22^. *C. elegans* make a useful model in elucidating the molecular mechanism of toxicity induction of ENMs in other organism due to the conservation of the basic stress response and molecular signaling pathways with mammals and humans^23^.

A few important signaling pathways, including insulin, c-Jun N-terminal kinase (JNK), apoptosis, and DNA damage signaling pathways have already been shown to be involved in the control of GO toxicity in nematodes^24^,^25^,^26^. The Wnt signaling pathway is one of the evolutionarily conserved signal transduction pathways, and is extensively utilized during the development of animals and humans^27^. In *C. elegans*, the Wnt signals are involved in controlling different aspects of development, including the proliferation, fate specification, and polarity^28^. Five Wnt ligands have been identified, and they are LIN-44, EGL-20, MOM-2, CWN-1, and CWN-2^28^. However, the role of Wnt signals in the regulation of GO toxicity is still unclear. In this study, the potential important role of Wnt signals in the regulation of GO toxicity in nematodes was investigated. Our results demonstrated that the Wnt signals differentially regulated the toxicity and translocation of GO. Out data also suggests that the Wnt signals might act through different mechanisms in the regulation of GO toxicity in nematodes.

## Results

### Effects of *cwn-1, cwn-2, mom-2, egl-20*, or *lin-44* mutation on GO toxicity

**Figure 1:**
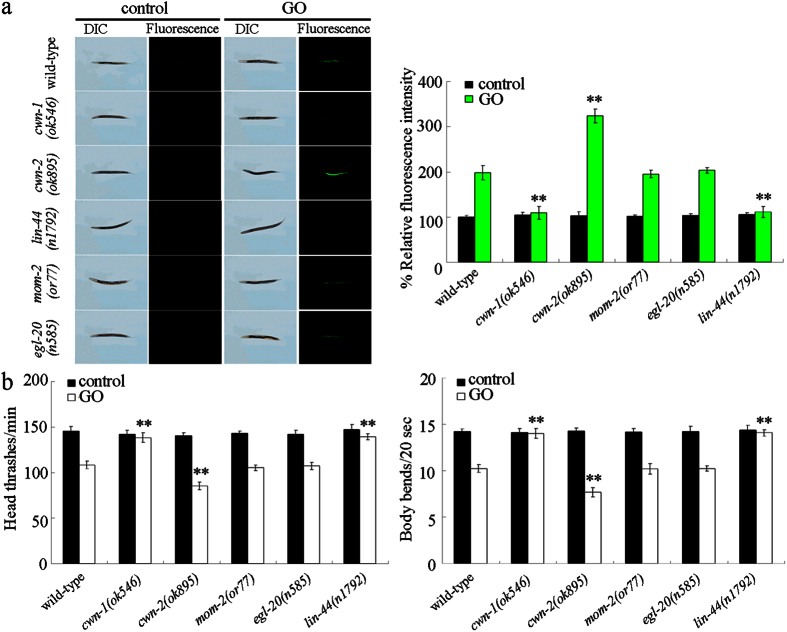
Effects of *cwn-1, cwn-2, mom-2, egl-20*, or *lin-44* mutation on GO toxicity in nematodes. (**a**) Effects of *cwn-1, cwn-2, mom-2, egl-20*, or *lin-44* mutation on GO toxicity in inducing intestinal ROS production in nematodes. (**b**) Effects of *cwn-1, cwn-2, mom-2, egl-20*, or *lin-44* mutation on GO toxicity in decreasing locomotion behavior in nematodes. GO exposure concentration was 100 mg/L. Prolonged exposure was performed from L1-larvae to young adults. Bars represent means ± SD. ^**^P < 0.01 *vs* wild-type.

In order to determine the role of CWN-1, CWN-2, MOM-2, EGL-20, or LIN-44 in the regulation of GO toxicity, we investigated the effects of *cwn-1, cwn-2, mom-2, egl-20*, or *lin-44* mutation on GO toxicity in nematodes. It had been previously reported that prolonged exposure to GO at concentrations of 1–100 mg/L decreased the locomotion behavior and resulted in the significant induction of intestinal ROS production in nematodes^19^. In this study, the 100 mg/L was chosen as the working concentration for GO. Typically, the *cwn-1, cwn-2, mom-2, egl-20*, or *lin-44* mutants do not show a significant production of intestinal reactive oxygen species (ROS), or an altered locomotion behavior (Fig. 1b). After GO (100 mg/L) exposure, we found that mutation of *cwn-1* or *lin-44* gene induced a resistant property to GO toxicity, whereas mutation of *cwn-2* gene induced a susceptible property to GO toxicity, using intestinal ROS production and locomotion behavior as the endpoints (Fig. 1). In contrast to these, mutation of *mom-2* or *egl-20* gene did not obviously influence the GO toxicity, as measured by the induction of intestinal ROS production and the decrease in locomotion behavior (Fig. 1). These results imply that CWN-1, CWN-2, and LIN-44 play an important role in the regulation of GO toxicity in nematodes.

### Genetic interactions of Wnt ligands in regulating GO toxicity

**Figure 2:**
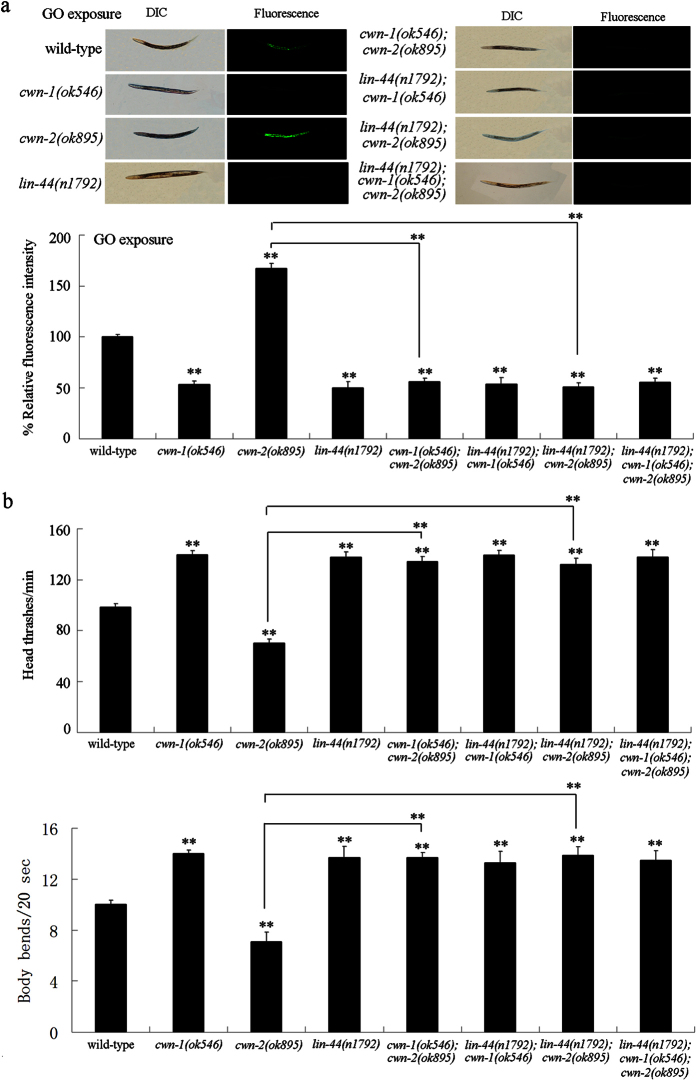
Genetic interactions of Wnt ligands in regulating GO toxicity in nematodes. (**a**) Genetic interactions of Wnt ligands in regulating GO toxicity in inducing intestinal ROS production in nematodes. (**b**) Genetic interactions of Wnt ligands in regulating GO toxicity in decreasing locomotion behavior in nematodes. GO exposure concentration was 100 mg/L. Prolonged exposure was performed from L1-larvae to young adults. Bars represent means ± SD. ^**^P < 0.01 *vs* wild-type (if not specially indicated).

Using double and triple mutants, the genetic interaction of the Wnt ligands, CWN-1, LIN-44, and CWN-2, in the regulation of GO toxicity in nematodes was further investigated. Under conditions without GO exposure, the double mutants (*cwn-1(ok546); cwn-2(ok895), lin-44(n1792); cwn-1(ok546), lin-44(n1792); cwn-2(ok895)*) and the triple mutant (*lin-44(n1792); cwn-1(ok546); cwn-2(ok895)*) do not exhibit significant intestinal ROS production, or an altered locomotion behavior (data not shown) (data not shown). Using intestinal ROS production and locomotion behavior as the endpoints, we found that mutation of *cwn-1* gene could inhibit the susceptibility of the*cwn-2(ok895)* mutant to GO toxicity, as shown by the induction of intestinal ROS production or the decreased locomotion behavior (Fig. 2). In summary, the GO (100 mg/L) exposed *cwn-1(ok546); cwn-2(ok895)* double mutant showed reduced intestinal ROS production and an increased locomotion behavior as compared to the GO (100 mg/L) exposed *cwn-2(ok895)* mutant (Fig. 2).

Similarly, using intestinal ROS production and locomotion behavior as the endpoints, we found that mutation of *lin-44* gene could suppress the susceptibility of the *cwn-2(ok895)* mutant to GO toxicity, as shown by the induction of intestinal ROS production or in decreasing locomotion behavior (Fig. 2). The GO (100 mg/L) exposed *lin-44(n1792); cwn-2(ok895)* double mutant exhibited a decrease in intestinal ROS production and an increased locomotion behavior, as compared with the GO (100 mg/L) exposed *cwn-2(ok895)* mutant (Fig. 2).

The data described above indicate that the *cwn-1(ok546)* and *lin-44(n1792)* mutants were resistant to GO toxicity, and that the locomotion behavior of the GO (100 mg/L) exposed *cwn-1(ok546)* or *lin-44(n1792)* mutant was similar to that of the wild-type nematodes that had not been exposed to GO. In order to examine the interaction between CWN-1 and LIN-44 in regulating of GO toxicity, the concentration of 1000 mg/L was selected as the working concentration for GO. Under normal conditions, the number of head thrashes per min for wild-type nematodes is approximately 140, and the number of body bends per 20 sec is approximately 14. After exposure to GO (1000 mg/L), despite the *cwn-1(ok546)* or the *lin-44(n1792)* mutant still being resistant to GO toxicity, both the number of head thrashes per min and the number of body bends per 20 sec were lower than those observed for the wild-type nematodes without the GO exposure (Fig. S1). The induction of intestinal ROS production and the locomotion behavior in the GO (1000 mg/L) exposed *lin-44(n1792); cwn-1(ok546)* double mutant were similar to what was observed for the GO (1000 mg/L) exposed *cwn-1(ok546)* mutant or the GO (1000 mg/L) exposed *lin-44(n1792)* mutant nematodes (Fig. S1). These results imply that CWN-1 and LIN-44 may function in the same genetic pathway in the regulation of GO toxicity.

The effects of the loss-of-function of three candidate Wnt ligand genes, *cwn-1, cwn-2*, and *lin-44*, on the GO toxicity in nematodes were further investigated. After GO (100 mg/L) exposure, the *lin-44(n1792); cwn-1(ok546); cwn-2(ok895)* triple mutant exhibited a similar induction of intestinal ROS production as well as locomotion behavior as compared to what was observed for the *cwn-1(ok546); cwn-2(ok895), lin-44(n1792); cwn-1(ok546)*, or *lin-44(n1792); cwn-2(ok895)* double mutant (Fig. 2). Therefore, Wnt signals may, as a whole, positively regulate the GO toxicity in nematodes.

### Distribution and translocation of GO in *cwn-1, cwn-2*, and *lin-44* mutant nematodes

**Figure 3:**
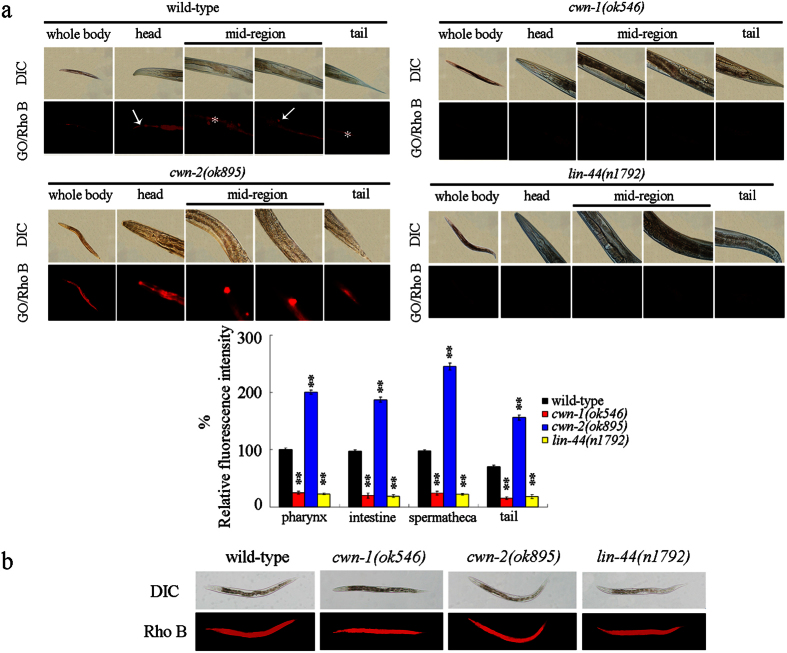
Distribution and translocation of GO in the body of wild-type, *cwn-1, cwn-2*, and *lin-44* mutant nematodes. (**a**) Distribution of GO/Rho B in the body of wild-type, *cwn-1, cwn-2*, and *lin-44* mutant nematodes. Arrowheads indicate pharynx in the head and spermatheca in the mid-region, respectively. Asterisks indicate intestine in the mid-region and tail, respectively. GO/Rho B exposure concentration was 100 mg/L. Bars represent means ± SD. ^**^P < 0.01 *vs* wild-type. (**b**) Distribution of Rho B in the body of wild-type, *cwn-1, cwn-2*, and *lin-44* mutant nematodes.

Considering the crucial role of bio-distribution and translocation of toxic ENMs in their potential toxicity formation^14^,^29^,^30^, the bio-distribution and translocation of GO in the *cwn-1, cwn-2*, and *lin-44* mutant nematodes was further examined by labeling GO with the molecular probe Rhodamine B (Rho B). After exposure, the GO/Rho B could be observed in both the primary targeted organs, such as the pharynx and the intestine, as well as the secondary targeted organs, such as the spermatheca in the wild-type nematodes (Fig. 3a). A large amount of GO/Rho B was also observed in the tail of the wild-type nematodes (Fig. 3a). Mutation of *cwn-2* gene enhanced the accumulation of GO/Rho B in the body of nematodes (Fig. 3a). A more pronounced accumulation of GO/Rho B was observed in the pharynx, the intestine, the spermatheca, and the tail of the *cwn-2(ok895)* mutant, as compared with what was observed in the wild-type nematodes (Fig. 3a). In contrast to this, mutation of *cwn-1* or *lin-44* obviously reduced the accumulation of GO/Rho B in the body of the nematodes (Fig. 3a). Only very weak signals of GO/Rho B were detected in the pharynx, the intestine, the spermatheca, and the tail in the *cwn-1(ok546)* or *lin-44(n1792)* mutant nematodes (Fig. 3a). Compared with the distribution of GO/Rho B, exposure to Rho B resulted in the relatively equable accumulation of fluorescence in the tissues of wild-type, *cwn-1(ok546), cwn-2(ok895)*, or *lin-44(n1792)* mutant nematodes (Fig. 3b). Therefore, mutation of *cwn-1, cwn-2*, or *lin-44* gene may affect the uptake of GO in the body of nematodes.

### Mutation of *cwn-1, cwn-2*, or *lin-44* gene altered the intestinal permeability in GO exposed nematodes

**Figure 4:**
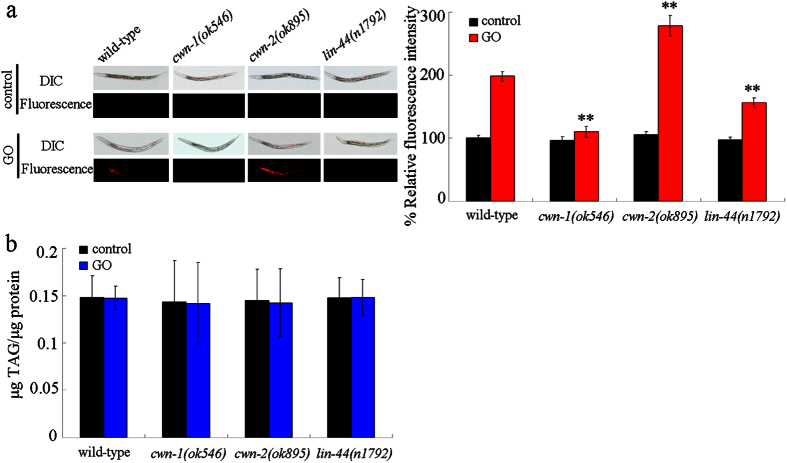
Effects of *cwn-1, cwn-2*, or *lin-44* mutation on intestinal permeability in GO exposed nematodes. (**a**) Comparison of relative fluorescence intensities of Nile Red in nematodes. (**b**) Comparison of triglyceride amount. GO exposure concentration was 100 mg/L. Prolonged exposure was performed from L1-larvae to young adults. Bars represent means ± SD. ^**^P < 0.01 *vs* wild-type.

The enhanced intestinal permeability in nematodes is one of the important cellular contributors for GO toxicity^19^. The lipophilic fluorescent dye, Nile Red, was used in order to stain the GO exposed wild-type, *cwn-1, cwn-2*, and *lin-44* mutant nematodes. Mutation of *cwn-1, cwn-2*, or *lin-44* gene did not alter the relative fluorescence intensity of Nile Red signal in intestine and the triglyceride content in nematodes that were not exposed to GO (Fig. 4). After GO (100 mg/L) exposure, mutation of *cwn-2* gene significantly increased the relative fluorescence intensity of Nile Red signal in the intestine as compared with the wild-type, and mutation of *cwn-1* gene significantly decreased the relative fluorescence intensity of Nile Red signal in the intestine as compared with wild-type (Fig. 4a). Meanwhile, mutation of *lin-44* gene only moderately decreased the relative fluorescence intensity of Nile Red signal in the intestine as compared with wild-type (Fig. 4a). Considering the fact that Nile Red can be used to label fat storage in nematodes^31^, we also investigated the triglyceride contents in the wild-type, *cwn-1, cwn-2*, and *lin-44* mutant nematodes. After exposure to GO (100 mg/L), the triglyceride content in the *cwn-1(ok546), cwn-2(ok895)*, or *lin-44(n1792)* mutants was similar to what was observed for the wild-type (Fig. 4b). Based on these data, we conclude the possibility that mutation of *cwn-1, cwn-2*, or *lin-44* gene may differentially influence the intestinal permeability in GO exposed nematodes.

### Mutation of *cwn-1*, or *lin-44* gene affected the defecation behavior in GO exposed nematodes

**Figure 5:**
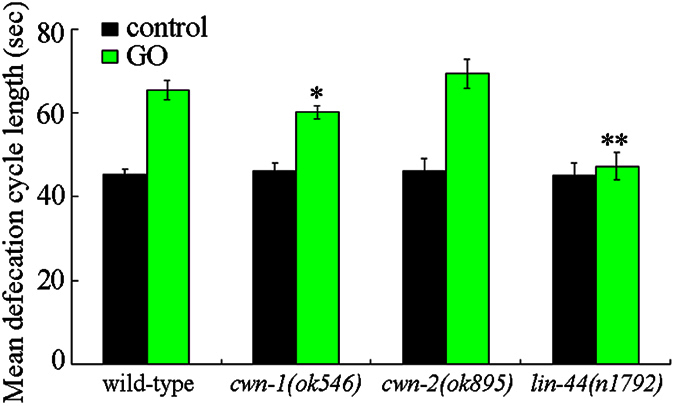
Effects of *cwn-1, cwn-2*, or *lin-44* mutation on mean defecation cycle length in GO exposed nematodes. GO exposure concentration was 100 mg/L. Prolonged exposure was performed from L1-larvae to young adults. Bars represent means ± SD. ^*^P < 0.05, ^**^P < 0.01 *vs* wild-type.

Another important cellular contributor to GO toxicity is the prolonged defecation cycle length in nematodes^19^. The endpoint of the mean defecation cycle length was used in order to reflect the defecation behavior in GO exposed wild-type, *cwn-1, cwn-2*, and *lin-44* mutant nematodes. Mutation of *cwn-1, cwn-2*, or *lin-44* gene did not obviously affect the mean defecation cycle length in nematodes without GO exposure (Fig. 5). After GO (100 mg/L) exposure, mutation of *cwn-2* gene did not significantly influence the mean defecation cycle length compared with wild-type nematodes (Fig. 5). In contrast, after GO (100 mg/L) exposure, mutation of *lin-44* gene significantly decreased the mean defecation cycle length compared with wild-type nematodes (Fig. 5). Meanwhile, after GO (100 mg/L) exposure, mutation of *cwn-1* gene only moderately decreased the mean defecation cycle length compared with wild-type nematodes (Fig. 5). Therefore, mutation of *cwn-1* and *lin-44* genes may affect the defecation behavior in GO exposed nematodes.

### Effect of GO exposure on expression patterns of genes encoding Wnt ligands

**Figure 6:**
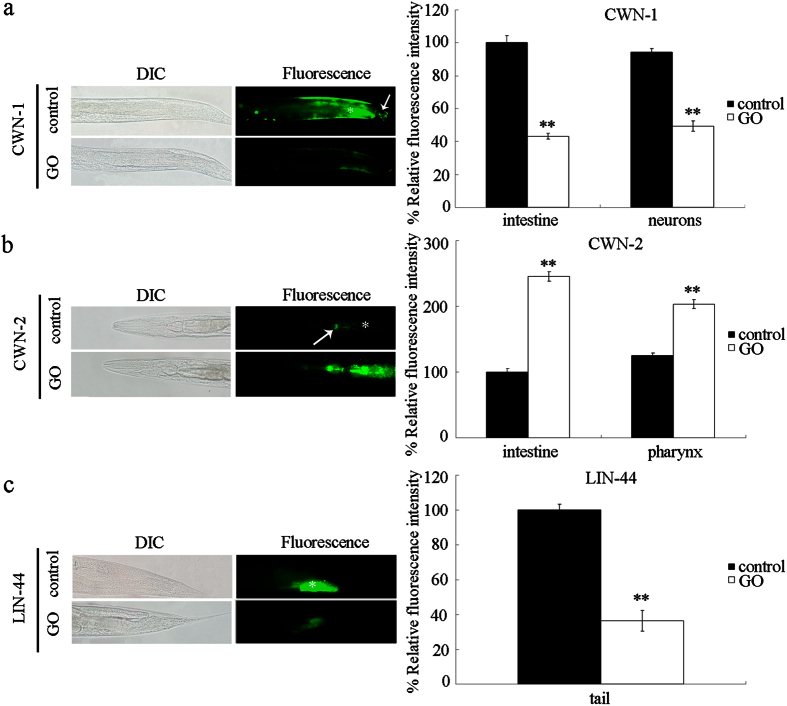
Effect of GO exposure on expression patterns of CWN-1, CWN-2, and LIN-44 in nematodes. (**a**) Effect of GO exposure on expression patterns of CWN-1 in nematodes. Arrowhead indicates the neurons, and asterisk indicates the intestine. (**b**) Effect of GO exposure on expression patterns of CWN-2 in nematodes. Arrowhead indicates the pharynx, and asterisk indicates the intestine. (**c**) Effect of GO exposure on expression patterns of LIN-44 in nematodes. Asterisk indicates the tail. GO exposure concentration was 100 mg/L. Prolonged exposure was performed from L1-larvae to young adults. Bars represent means ± SD. ^**^P < 0.01 *vs* control.

In *C. elegans*, CWN-1 is expressed in the neurons and the intestine during the development^32^. CWN-2 is predominantly expressed in the pharynx and the intestine^32^. LIN-44 is only expressed in the posterior of the animal, and is present in the tail hypodermis, *hyp8, hyp9, hyp10*, and *hyp11* cells^33^. After exposure, we observed that GO (100 mg/L) significantly decreased the expression of CWN-1 in the intestine and the neurons (Fig. 6a), increased the expression of CWN-2 in the intestine and the pharynx (Fig. 6b), and decreased the expression of LIN-44 in the tail of nematodes (Fig. 6c).

## Discussion

It has been previously reported that the activation of toll-like receptor 4 (TLR4) signaling and the subsequent autocrine TNF-α production may regulate the GO toxicity in macrophages^34^. Exposure to GO could also induce cytotoxicity by activating the genuine autophagy^35^. Our previous studies in *C. elegans* have suggested that the insulin, JNK, apoptosis, and DNA damage signaling pathways are also involved in the control of GO toxicity^24^,^25^,^26^. In this study, we observed that exposure to GO could affect the expression patterns of three Wnt ligands, CWN-1, CWN-2, and LIN-44, in nematodes (Fig. 6), supporting the further involvement of these Wnt signals in the control of GO toxicity in organisms.

It has been shown in *C. elegans* that LIN-44 regulates the polarity of asymmetric cell division, cell fate specification, axon and dendrite outgrowth, and positions the neuromuscular connectivity^36^,^37^,^38^,^39^,^40^. CWN-1 regulates the cell competence, cell migration, asymmetric cell division, cell fate specification, cell polarity, and axon termination^41^,^42^,^43^,^44^,^45^. CWN-2 regulates the cell migration, cell polarity, cell fate specification, synaptic plasticity, neurite outgrowth, and axon termination^41^,^43^,^44^,^45^,^46^,^47^,^48^,^49^. More recently, studies have shown that all five Wnt ligands, including LIN-44, CWN-1, and CWN-2, are expressed during the development from larvae to the adult stage, and that LIN-44 is beneficial for a long lifespan^33^. Meanwhile, the thermo-tolerance and resistance to oxidative stress during aging were not significantly affected by any of the Wnt ligand mutants^33^. However, in this study, we found that mutation of *cwn-1* or *lin-44* gene caused a resistant property to GO toxicity, whereas mutation of *cwn-2* gene resulted in a susceptible property to GO toxicity (Fig. 1). In summary, in regards to controlling GO toxicity, CWN-1, CWN-2, or LIN-44 alone were shown to be result in noticeable effects and functions in nematodes. These results support an important role of certain Wnt signals in the regulation of stress responses. Nevertheless, not all Wnt signals are required for the control of stress response in organisms. Additionally, our results suggest that Wnt signals may regulate different stress responses using different mechanisms.

Among the three candidate Wnt ligands, we found that CWN-1 or LIN-44 could act antagonistically with CWN-2 in the regulation of GO toxicity in nematodes. Mutation of *cwn-1* or *lin-44* gene inhibited the GO toxicity susceptibility of the *cwn-2(ok895)* mutant (Fig. 2), suggesting that CWN-2 can suppress the function of CWN-1 or LIN-44 in the regulation of GO toxicity. These results suggest that different Wnt signals can act antagonistically regulate GO toxicity. The Wnt signals of CWN-1 and LIN-44 were shown to have the opposite function to CWN-2 in the regulation of GO toxicity in nematodes.

In this study, we observed that the induction of intestinal ROS production and the locomotion behavior in GO exposed *lin-44(n1792); cwn-1(ok546)* mutants were similar to what was observed in the GO exposed *cwn-1(ok546)* or *lin-44(n1792)* mutants (Fig. S1), which implies that these two Wnt ligands, CWN-1 and LIN-44, may act in the same genetic pathway in the regulation of GO toxicity. Addtionally, we found that triple mutations of *cwn-1, cwn-2*, and *lin-44* genes resulted in a resistant property to GO toxicity, as was observed for the *cwn-1(ok546); cwn-2(ok895), lin-44(n1792); cwn-1(ok546)*, or *lin-44(n1792); cwn-2(ok895)* double mutants (Fig. 2), which suggests that *cwn-1* and *lin-44* are epistatic to *cwn-2* in the regulation of GO toxicity. These results suggest the existence of CWN-2-CWN-1/LIN-44 signaling cascade in the regulation of GO toxicity in nematodes.

In this study, we found that mutation of *cwn-1, cwn-2*, or *lin-44* gene could noticeably alter the uptake of GO in the body of nematodes. Mutation of *cwn-1* or *lin-44* gene suppressed the accumulation of GO in the body of nematodes; however, mutation of *cwn-2* gene enhanced the accumulation of GO in the body of nematodes (Fig. 3a). Therefore, CWN-1, CWN-2, and LIN-44 may regulate both the toxicity and uptake of GO in nematodes. One of the important cellular mechanisms for the observed differences in the toxicity and translocation of GO in *cwn-1, cwn-2*, or *lin-44* mutants is that the mutations in the *cwn-1* or *lin-44* gene were shown to be helpful in the maintenance of normal intestinal permeability in GO exposed nematodes, and that mutations in the *cwn-2* gene enhanced the intestinal permeability in GO exposed nematodes (Fig. 4). Thus, the enhanced intestinal permeability observed for the *cwn-2* mutant may strengthen GO uptake, whereas the decreased intestinal permeability in the *cwn-1* or *lin-44* mutants may suppress the GO uptake. Another important cellular mechanism for the observed differences in the toxicity and translocation of GO in the *cwn-1, cwn-2*, or *lin-44* mutants is that mutations in the *cwn-1* or *lin-44* genes could significantly decrease the mean defecation cycle length in the GO exposed nematodes (Fig. 5), implying that the *cwn-1* and *lin-44* mutants may be relatively faster in excluding GO from the body as compared with the GO exposed wild-type nematodes.

Our results suggest that the *cwn-1* and *cwn-2* genes might regulate the intestinal permeability of GO exposed nematodes (Fig. 5). In contrast, the *lin-44* gene might regulate the defecation behavior of GO exposed nematodes (Fig. 6). The moderate alteration in the intestinal permeability observed for the GO exposed *lin-44(n1792)* mutant may be largely due to the reduced accumulation of GO in the intestine and the decreased mean defecation cycle length in the GO exposed *lin-44(n1792)* mutant nematodes. The moderate alteration in the mean defecation cycle length observed for the GO exposed *cwn-1(ok546)* mutant may be largely due to the reduced accumulation of GO in the tail region and the decreased intestinal permeability of the GO exposed *cwn-1(ok546)* mutant nematodes. Therefore, the Wnt ligands, CWN-1, CWN-2, and LIN-44, may differentially regulate the toxicity and translocation of GO through different cellular mechanisms in nematodes.

In conclusion, the present study investigated the molecular mechanism of Wnt signals in the control of GO toxicity in nematodes. Our results demonstrated that the Wnt ligands CWN-1, CWN-2, and LIN-44 are involved in controlling the toxicity and translocation of GO. In controlling GO toxicity, CWN-1 or LIN-44 functioned antagonistically with CWN-2. The loss-of-function mutations of all three of these Wnt ligands induced a resistance to GO toxicity in nematodes. Moreover, we found that these three Wnt ligands may regulate the GO toxicity through different mechanisms. Our results will be helpful in understanding the crucial role of Wnt signals in the regulation of GO toxicity in organisms.

## Methods

### Preparation of GO

GO was prepared from the natural graphite powder according to a modified Hummer’s method^50^. In a 250-mL flask, graphite (2 g) and sodium nitrate (1 g) were first added. Concentrated H_2_SO_4_ (50 mL) was added on ice. KMnO_4_ (7 g) was then added. When the temperature of mixture reached to 35 °C, H_2_O (90 mL) was slowly dripped into the paste. After stirring the diluted suspension at 70 °C for 15 min, the suspension was treated with a mixture of 7 mL of 30% H_2_O_2_ and 55 mL of H_2_O. The warm suspension was filtered to obtain a yellow-brown filter cake, which was washed with 3% HCl, followed by drying at 40 °C for 24 h. GO was obtained after ultrasonication of as-made graphite oxide in water for 1 h.

### Characterization of GO

In this study, GO was characterized by atomic force microscopy (AFM, SPM-9600, Shimadzu, Japan), Raman spectroscopy, and zeta potential. Based on the AFM assay, the GO thickness was approximately 1.0 nm in topographic height, corresponding to the property of one layer (Fig. S2a). Sizes of GO in K-medium after sonication (40 kHz, 100 W, 30 min) were mainly in the range of 40–50 nm (Fig. S2b). Raman spectroscopy was analyzed using a 632 nm wavelength excitation (Renishaw Invia Plus laser Raman spectrometer, Renishaw, UK). Raman spectroscopy assay demonstrated the existence of G band at 1589 cm^−1^ and D band at 1350 cm^−1^ (Fig. S2c). Zeta potential was analyzed by dynamic light scattering (DLS) using a Nano Zetasizer (Malvern Instrument Ltd., UK). Zeta potential of GO (100 mg/L) in K-medium was −21.8 ± 2.4 mV.

### *C. elegans* strains and exposure

Nematodes used were wild-type N2, mutants of *cwn-1(ok546), cwn-2(ok895), lin-44(n1792), egl-20(n585), mom-2(or77), lin-44(n1792); cwn-2(ok895), cwn-1(ok546); cwn-2(ok895), lin-44(n1792); cwn-1(ok546)*, and *lin-44(n1792); cwn-1(ok546); cwn-2(ok895)*, and transgenic strains of *osEx397*[P*cwn-1-cwn-1::Venus*], *osEx393*[P*cwn-2-cwn-2::Venus*], and *ksEx29*[*lin-44::GFP*]. The *cwn-1(ok546), cwn-2(ok895)*, and *lin-44(n1792)* mutants are loss-of-function mutants. Some of the used strains were from *Caenorhabditis* Genetics Center (funded by NIH Office of Research Infrastructure Programs (P40 OD010440)). Nematodes were maintained on nematode growth medium (NGM) plates seeded with *Escherichia coli* OP50 at 20 °C^17^, and lysed with a bleaching mixture (0.45 M NaOH, 2% HOCl) after washing off the plates into the centrifuge tubes. The age synchronous L1-larvae populations were prepared as described previously^51^.

### Exposure and toxicity assessment

The stock solution of GO (1 mg/mL) in K medium was sonicated for 30 min (40 kHz, 100 W). The GO at the working concentrations (100 and 1000 mg/L) were prepared by diluting the stock solution with K medium. Prolonged exposure to GO from L1-larvae to young adults was performed in 12-well sterile tissue culture plates at 20 °C in the presence of food (OP50). After GO exposure, the exposed nematodes were used for the toxicity assessment with the intestinal ROS production and the locomotion behavior as the endpoints.

The endpoint of intestinal ROS production can reflect the functional state of the primary targeted organ of intestine in nematodes^52^. Intestinal ROS production was analyzed as described previously^53^,^54^. After GO exposure, the examined nematodes were transferred to 1 μM of 5′,6′-chloromethyl-2′,7′-dichlorodihydro-fluorescein diacetate (CM-H_2_DCFDA; Molecular Probes) solution to incubate for 3 h in the dark. After labeling, the nematodes were mounted on a 2% agar pad for the observation and examination at 488 nm of excitation wavelength and 510 nm of emission filter under a laser scanning confocal microscope (Leica, TCS SP2, Bensheim, Germany). Relative fluorescence intensity of ROS signals in the intestine was semi-quantified and expressed as the relative fluorescence units (RFU). Ten nematodes were examined per treatment, and seven replicates were performed.

The endpoint of locomotion behavior can be used to reflect the functional state of motor neurons in nematodes^55^. The head thrash and body bend were selected to evaluate the locomotion behavior in nematodes. The head thrash and body bend were analyzed under the dissecting microscope by eye as described previously^56^,^57^. In *C. elegans*, a head thrash is defined as a change in the direction of bending at the mid body, and a body bend is defined as a change in the direction of the part of the nematodes corresponding to the posterior bulb of the pharynx along the *y* axis, assuming that nematode was traveling along the *x* axis^56^. Twenty nematodes were examined per treatment, and seven replicates were performed.

### Distribution and translocation of GO

To investigate the *in vivo* translocation and distribution of GO, Rho B was loaded on the GO by mixing Rho B solution (1 mg/mL, 0.3 mL) with aqueous GO suspension (0.1 mg/mL, 5 mL) as described^58^. The unbound Rho B was removed by dialysis against the distilled water over 72 h. The finally resulting GO/Rho B was stored at 4 °C. Nematodes were incubated with GO/Rho B for 3 h, and washed with three times of M9 buffer. After the exposure, the nematodes were observed and analyzed under a laser scanning confocal microscope (Leica, TCS SP2, Bensheim, Germany). Rho B staining alone was used to serve as the control.

### Nile Red staining

The Nile Red staining method was performed as described previously^30^. The Nile Red (Molecular Probes, Eugene, OR) was dissolved in the acetone to prepare a 0.5 mg/mL stock solution and stored at 4 °C. The stock solution was freshly diluted in 1 x PBS buffer to obtain the working solution at the concentration of 1 mg/mL for the use of Nile Red staining. Twenty nematodes were examined per treatment, and five replicates were performed.

### Analysis of triglyceride content

Lipid was extracted in nematodes as described previously^59^. The triglyceride content was measured using an enzymatic kit (Wako Triglyceride E-test, Wako Pure Chemical Ltd, Osaka, Japan). Ten replicates were performed.

### Defecation behavior analysis

The mean defecation cycle length was analyzed as described^60^. Individual nematodes were examined for their fixed number of cycles. A cycle period was defined as the interval between initiations of two successive posterior body-wall muscle contraction steps. Six nematodes were examined per treatment, and twenty replicates were performed.

### Statistical analysis

Data in this article were expressed as means ± standard deviation (SD). Statistical analysis was performed using SPSS 12.0 software (SPSS Inc., Chicago, USA). Differences between groups were determined using analysis of variance (ANOVA), and probability levels of 0.05 and 0.01 were considered statistically significant. Graphs were generated using Microsoft Excel software (Microsoft Corp., Redmond, WA).

## Additional Information

**How to cite this article:** Zhi, L. *et al*. Wnt Ligands Differentially Regulate Toxicity and Translocation of Graphene Oxide through Different Mechanisms in *Caenorhabditis elegans. Sci. Rep.* **6**, 39261; doi: 10.1038/srep39261 (2016).

**Publisher's note:** Springer Nature remains neutral with regard to jurisdictional claims in published maps and institutional affiliations.

## Supplementary Material

Supporting Information
