# Supplementary material for: Wnt Ligands Differentially Regulate Toxicity and Translocation of Graphene Oxide through Different Mechanisms in Caenorhabditis elegans
Source: Sci Rep. 2016 Dec 13;6:39261. doi: 10.1038/srep39261 (PMC5153639; doi:10.1038/srep39261)
Supplement: Supporting Information [file srep39261-s1.doc]

**Wnt Ligands Differentially Regulate Toxicity and Translocation of Graphene Oxide through Different Mechanisms in *Caenorhabditis elegans***

Lingtong Zhi, Mingxia Ren, Man Qu, Hanyu Zhang & Dayong Wang^*^

Key Laboratory of Environmental Medicine Engineering in Ministry of Education, Medical School, Southeast University, Nanjing 210009, China

^*^Correspondence and requests for materials should be addressed to D.W. (email: [dayongw@seu.edu.cn](mailto:dayongw@seu.edu.cn)).

**Supporting Information:**


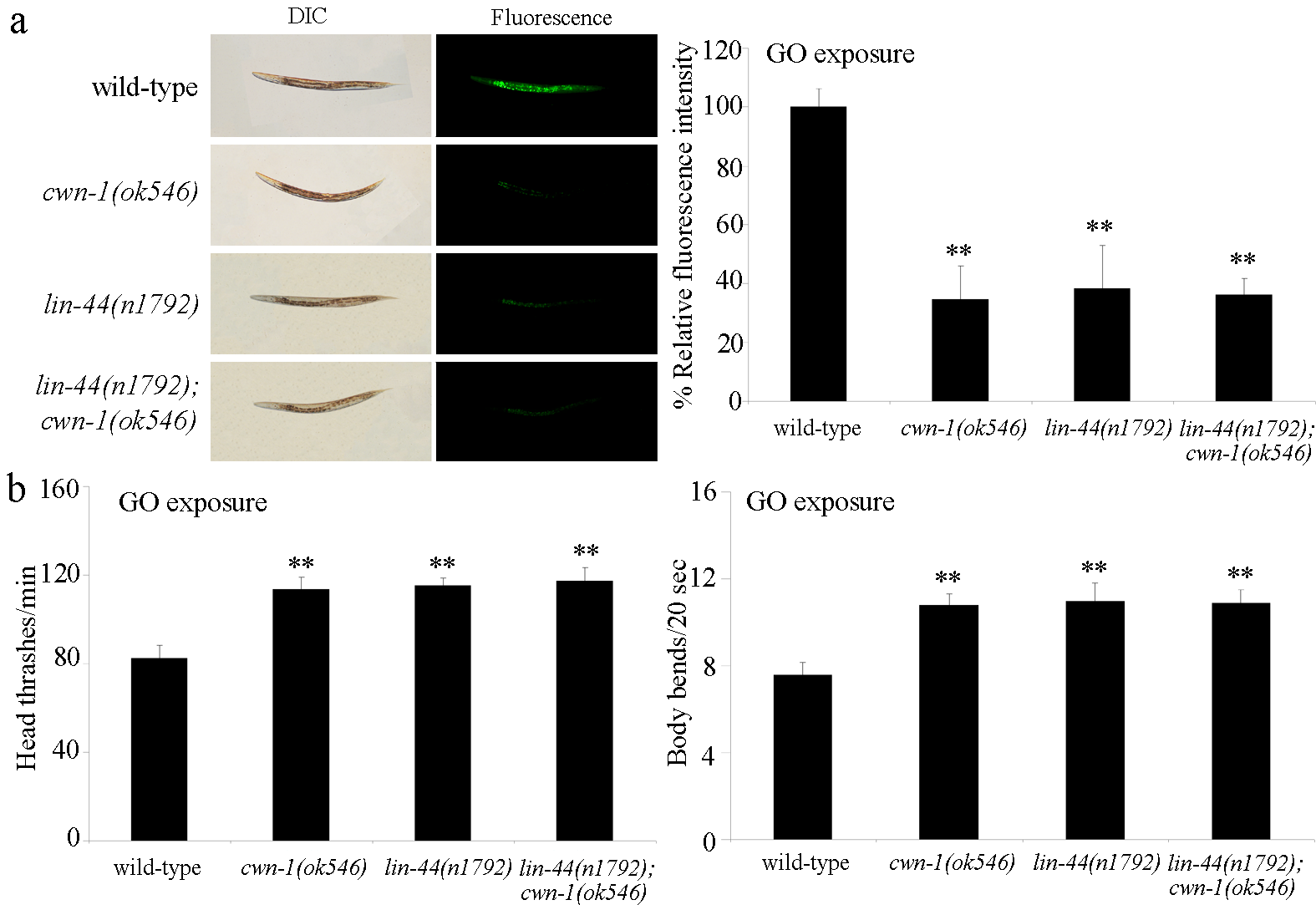


**Figure S1.** **Genetic interaction between CWN-1 and LIN-44 in regulating GO toxicity.**  (**a**) Genetic interaction between CWN-1 and LIN-44 in regulating GO toxicity in inducing intestinal ROS production. (**b**) Genetic interaction between CWN-1 and LIN-44 in regulating GO toxicity in decreasing locomotion behavior. GO exposure concentration was 1000 mg/L. Prolonged exposure was performed from L1-larvae to young adults. Bars represent means ± SD. ^**^*P* < 0.01 *vs* wild-type.


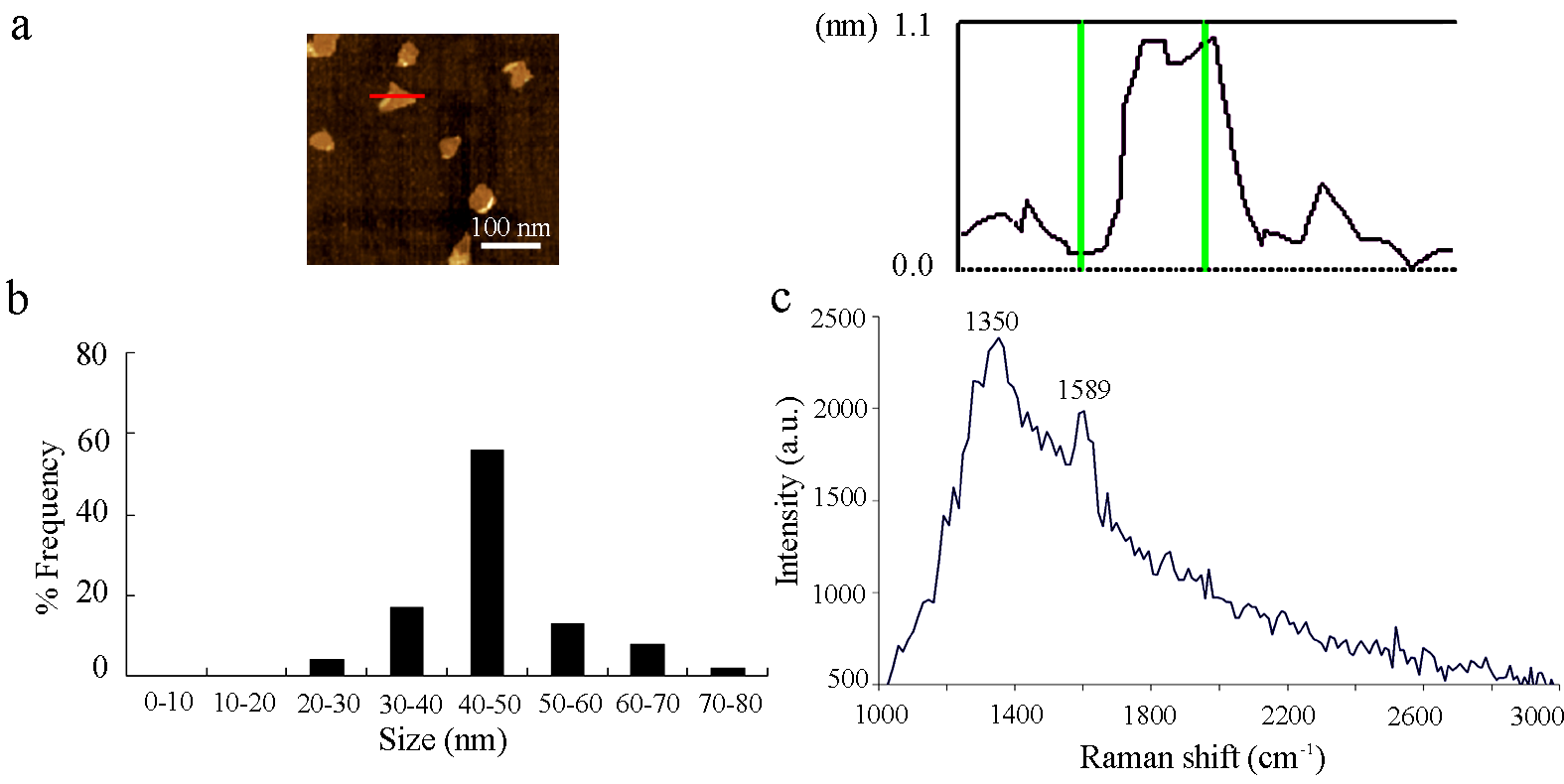


**Figure S2.** **Physiochemical properties of GO.** (**a**) AFM analysis of GO after sonication. (**b**) Size distribution of GO after sonication. (**c**) Raman spectrum of GO.
